# 433. Impact of Antibiotics on the Gut Microbiota: Comparisons Using Model-Based Analyses

**DOI:** 10.1093/ofid/ofae631.147

**Published:** 2025-01-29

**Authors:** Carlos Olivares, Jean de Gunzburg, Etienne Ruppe, Antoine Andremont, Tanguy Corbel, Stéphanie Ferreira, Jérémie Guedj, Charles Burdet

**Affiliations:** Université Paris Cité, IAME, Inserm, F-75018, Paris, France, Paris, Ile-de-France, France; Da Volterra, Paris, France, Paris, Ile-de-France, France; Université Paris Cité; Université Paris Cité, IAME, Inserm, F-75018, Paris, France, Paris, Ile-de-France, France; Da Volterra, Paris, France, Paris, Ile-de-France, France; Da Volterra, Paris, France, Paris, Ile-de-France, France; Université Paris Cité, IAME, Inserm, F-75018, Paris, France, Paris, Ile-de-France, France; Université Paris Cité, IAME, Inserm, F-75018, Paris, France, Paris, Ile-de-France, France

## Abstract

**Background:**

Antibiotic (AB) treatments shift the gut microbiota composition according to their spectrum of activity, posology and intestinal excretion rate. We compared the impact of various ABs on gut microbiota bacterial diversity.

**Methods:**

We used data from 2 RCTs, DAV-132-CL1004 and DAV-132-CL1006 (sponsor Da Volterra), where respectively 143 and 144 healthy volunteers (HV) were randomly assigned to receive a 5-day IV treatment with moxifloxacin (MXF, 400mg OAD, CL1004) or with ceftriaxone (CRO,1 g once a day), ceftazidime/avibactam (CEF/AVI, 2g/0.5g every 8 hours (q8h)), piperacillin/tazobactam (PIP/TZB, 4g/0.5g q8h) (CL1006). Some HV were also treated for 7 days with several doses of DAV132, a charcoal-based adsorbent that captures AB residuals in the late ileum and colon (2g-36g/day). Plasma and fecal concentrations of AB were measured longitudinally. Gut microbiota bacterial diversity was determined by 16S rRNA gene profiling. A pharmacokinetic (PK) model was adapted to predict plasma and feces AB concentrations over time, and a pharmacodynamic (PD) model of indirect response was used to characterize the effects of fecal drug on the bacterial diversity. In the parameter asymptotic distribution we simulated 1000 PK and PD profiles for each antibiotic with various treatment durations (3 to 14 days) and computed several PK & PD indices.

**Results:**

Following a 5-day treatment, the median [IQR] time for AB fecal concentrations to decrease below the limit of quantification after the last administration varied between antibiotics: CRO, 5.1 days [3.5;7.0]; CEF, 5.0 [3.2;8.5]; PIP, 2.5 [1.4;5.6]; and MXF, 11.5 [10.0;13.0].

The global impact on the diversity was highest for MXF and lowest for CRO: median AUC of the change from baseline of the Shannon index (SI) between baseline and day 42 of 13.2 [7.9;20.4] for MXF, 10.9 [0.6;20.4] for PIP, 10.1 [4.6;18.3] for CEF and 2.1 [0.8;5.0] for CRO. The maximal variation from baseline of the SI was observed for PIP (-27.3% [-37.0%;-1.9%]).

Impact increased for all AB when longer treatment durations were simulated.

**Conclusion:**

AB alter the intestinal microbiota diversity to various degrees, both within and between AB families. Such studies comparing the impact of AB on the gut microbiota may help in driving stewardship.

**Disclosures:**

**All Authors**: No reported disclosures

